# Thermal, Mechanical and Dielectric Properties of Polyimide Composite Films by In-Situ Reduction of Fluorinated Graphene

**DOI:** 10.3390/molecules27248896

**Published:** 2022-12-14

**Authors:** Yuyin Zhang, Tian Hu, Rubei Hu, Shaohua Jiang, Chunmei Zhang, Haoqing Hou

**Affiliations:** 1 College of Chemistry and Chemical Engineering, Jiangxi Normal University, Nanchang 330022, China; 2 Jiangsu Co-Innovation Center of Efficient Processing and Utilization of Forest Resources, International Innovation Center for Forest Chemicals and Materials, College of Materials Science and Engineering, Nanjing Forestry University, Nanjing 210037, China; 3 Institute of Materials Science and Devices, School of Materials Science and Engineering, Suzhou University of Science and Technology, Suzhou 215009, China

**Keywords:** polyimide, fluorinated graphene, composites, dielectric properties

## Abstract

Materials with outstanding mechanical properties and excellent dielectric properties are increasingly favored in the microelectronics industry. The application of polyimide (PI) in the field of microelectronics is limited because of the fact that PI with excellent mechanical properties does not have special features in the dielectric properties. In this work, PI composite films with high dielectric properties and excellent mechanical properties are fabricated by in-situ reduction of fluorinated graphene (FG) in polyamide acid (PAA) composites. The dielectric permittivity of pure PI is 3.47 and the maximum energy storage density is 0.664 J/cm^3^ at 100 Hz, while the dielectric permittivity of the PI composite films reaches 235.74 under the same conditions, a 68-times increase compared to the pure PI, and the maximum energy storage density is 5.651, a 9-times increase compared to the pure PI films. This method not only solves the problem of the aggregation of the filler particles in the PI matrix and maintains the intrinsic excellent mechanical properties of the PI, but also significantly improves the dielectric properties of the PI.

## 1. Introduction

With the progress of science and technology and technological innovation, electronic components are developing towards integration, miniaturization and high speed [1,2,3,4]. Although traditional ceramic materials have excellent dielectric properties, they struggle to meet the new demands of the development of the electronics industry due to their great brittleness, high density, severe losses and difficulty to process [5,6,7]. Polymer materials have the advantages of excellent flexibility, lightweight, tensile resistance and good processability [8,9,10,11,12,13,14,15], but their dielectric permittivity is generally low [16,17]. Therefore, polymer-matrix dielectric composites are widely studied and applied, as they can combine the excellent properties of each component [18,19,20,21,22,23,24].

Polyimide (PI) with a large number of imide rings in the main chain can be obtained by poly-condensation and imidization of equimolar amounts of dianhydride and diamine [25,26]. PI possesses excellent mechanical, thermal and optical properties, as well as good processability, and can therefore be used as the matrix for high dielectric composites [27]. The dielectric properties of PI matrix composites depend mainly on the type and dispersion of the filler. When inorganic ceramic particles, such as BaTiO_3_ and CaCu_3_Ti_4_O_12_ (CCTO) are used as filler, the PI composites have high dielectric permittivity, low dielectric loss and the dielectric properties of the composites are less dependent on temperature and frequency, but there are problems with inhomogeneous filler dispersion and severe loss of the mechanical properties of the PI [27,28,29,30,31,32]. With organic fillers, such as polyvinylidene fluoride (PVDF) and polysulfone (PSF), the resulting materials possess better distribution of each component, but the improvement of the dielectric properties is not obvious [33,34,35,36]. When conductive materials, such as carbon nanotubes (CNTs), graphene oxide (GO) and Ag are used as filler, better enhancement can be obtained at lower ratios, but there is difficulty in dispersing the filler particles and the dielectric loss of the composite increases significantly near the percolation threshold [37,38,39,40,41,42,43,44]. Therefore, it is still highly desired to search for good fillers, which can homogeneously disperse into the PI matrix, giving rise to the enhancement of both dielectric properties and mechanical properties.

Graphene is a carbon material with a single layer of two-dimensional honeycomb crystal structure with tightly stacked carbon atoms connected by SP^2^ hybridization, and has excellent properties, such as high electrical conductivity, high strength and high thermal conductivity [45,46]. Owing to its wide range of applications in physics, electronics and materials science, graphene is considered as a revolutionary material for the future [47,48]. Due to the huge van der Waals force between graphene sheets, they tend to be combined with each other and are difficult to be dispersed in polymers [49,50]. Fluorinated graphene (FG) is a new type of carbon material formed by fluorinating some or all of the carbon atoms in the graphene sheets [51,52]. As a new derivative of graphene, FG maintains the high strength properties of graphene, while introducing novel interfacial and physicochemical properties, such as reduced surface energy and enhanced hydrophobicity, arising from the introduction of fluorine atoms [53,54,55]. At the same time, FG also exhibits excellent properties, such as high temperature resistance, wear resistance and corrosion protection [56,57]. Due to the low polarization rate and the strong electronegativity of the fluorine atoms, the surface energy between the carbon sheets can be reduced, resulting in an easier dispersion of FG in the polymer.

In this work, FG is used as a filler to prepare PI composite films with ultra-high dielectric permittivity and low dielectric loss. PI composites are fabricated by in-situ reduction of FG in PAA composites. Compared to other types of PI composite films, this method not only solves the problem of aggregation of the filler particles in the PI matrix and maintains the intrinsic excellent mechanical properties of the PI, but also significantly improves the dielectric properties of the PI. Dispersed in the PI matrix uniformly, graphene sheets with excellent electrical conductivity can form a large number of micro-capacitance structures with each other, thus significantly improving the dielectric properties of the PI composite films.

## 2. Results and Discussion

### 2.1. Characterization of PI Composite Films

**Figure 1:**
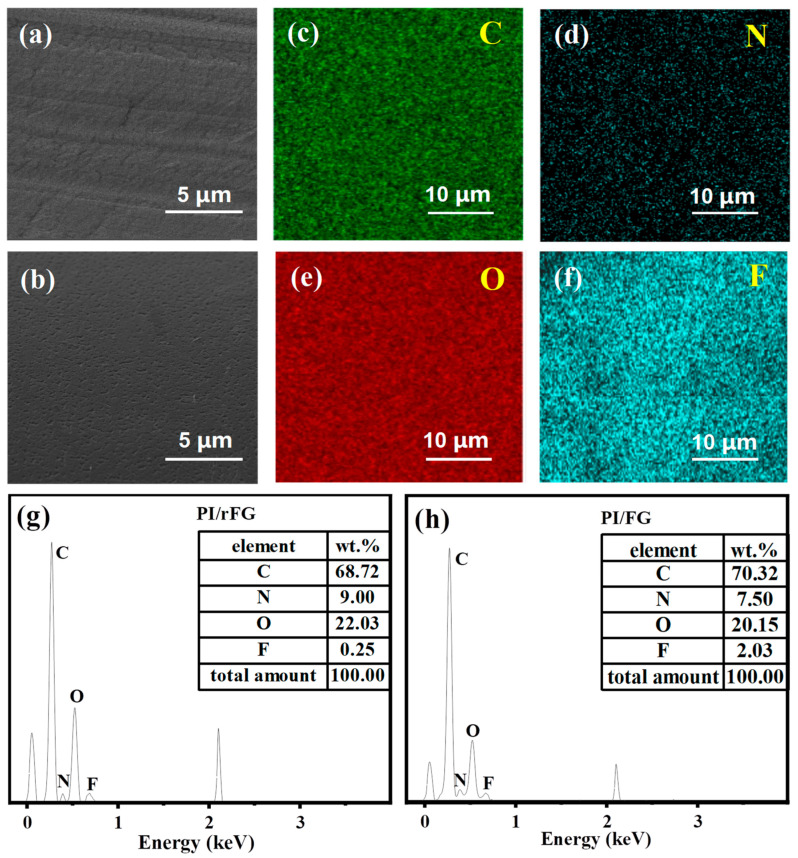
The characterization of PI composite films. (**a**) SEM image of the interface of the PI films; (**b**) SEM image of the interface of the PI/rFG-8 wt. % composite films; (**c**–**f**) element mappings of the PI/rFG-8% composite films; (**g**) the EDS of the PI/rFG-8% composite films; (**h**) the EDS of the PI/FG composite films with 8 wt. % FG of added without PEG.

**Figure 2:**
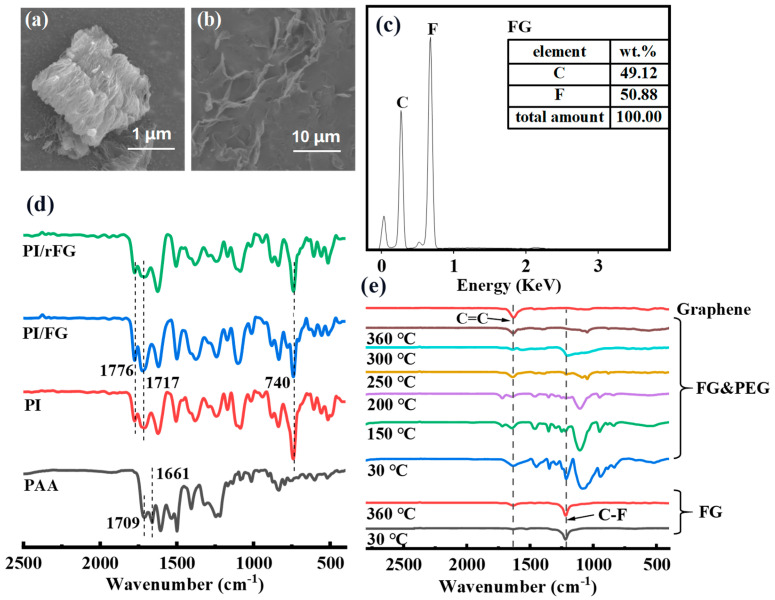
The characterization of the FG, PEG–FG and PI composite films. (**a**,**b**) the SEM image of the FG; (**c**) the EDS of the FG; (**d**) the FT-IR spectra of the PAA, PI, PI/FG-8% and PI/rFG-8% composite films; (**e**) the FT-IR spectra of graphene, FG–PEG and FG.

Inorganic materials added to PI as fillers often have the problem of inhomogeneous dispersion, causing a great loss of mechanical properties. The problem of inhomogeneous filler dispersion was solved by in-situ reduction of FG in PAA/FG–PEG composites. As shown in Figure 1a, the surface of the pure PI is smooth. When the FG–PEG addition ratio was 8 wt. %, the surfaces of the PI/rFG composite films were smooth compared to the pure PI and there was no accumulation of filler particle (Figure 1b). Other proportions of PI/rFG also had no accumulation of filler particle. As shown in Figure 2, FG was relatively chemically stable and remained stable at 360 °C. The prepared FG–PEG–DMAc mixture was annealed at different temperatures. When the temperature exceeded 250 °C, the absorption peak disappeared at 1220 cm^−1^ (C-F) and appeared at 1630 cm^−1^ (C=C), indicating that the FG was reduced. Figure 1c–f clearly shows that the composites contained elements of C, N, O and F. The elemental analysis and SEM images of the FG are shown in Figure 2. FG with a lamellar structure contains only two elements, C (49.12 wt. % or 7.255 g/mol) and F (50.88 wt. % or 7.515 g/mol). Without the addition of PEG, the PI/FG still possessed a relatively higher F amount of 2.03 wt. %, while the amount of F in the composite was only 0.25 wt. % when using PEG as a reducing agent (Figure 1g,h). The significant decrease of F content in the composite film indicates the reduction of FG to rFG.

The properties of the PI composite films are related to various factors, such as the degree of imidization, the dispersion of the filler particles, the components, etc. The process of forming PI by dehydrating PAA into a ring is called imidization. As shown in Figure 2, the carboxyl (1709 cm^−1^) and amide (1661 cm^−1^) absorption peaks in PAA are transformed into asymmetric stretching vibration peaks (1776 cm^−1^), symmetric stretching vibration peaks (1717 cm^−1^) and bending vibration absorption peaks (740 cm^−1^) of the imide structure, indicating that the PAA has been fully formed into PI. The characteristic absorption peak of graphene in the PI/rFG composite films is not obvious due to the influence of PI.

### 2.2. Thermal Properties and Mechanical Properties

**Figure 3:**
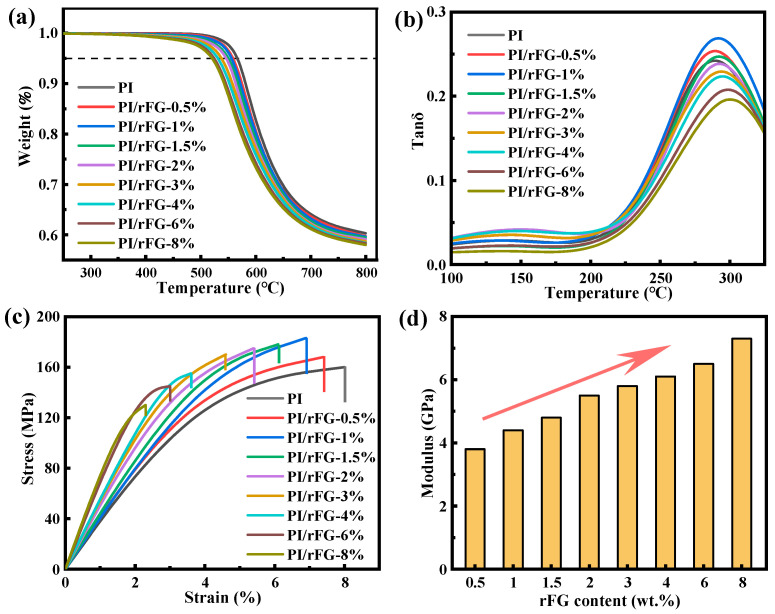
The thermal property and mechanical properties of the PI/rFG composite films. (**a**) Thermogravimetric analysis (TGA); (**b**) dynamic mechanical analysis (DMA); (**c**) stress–strain curve of the PI/rFG composite films; (**d**) modulus corresponding to the rFG content.

**Figure 4:**
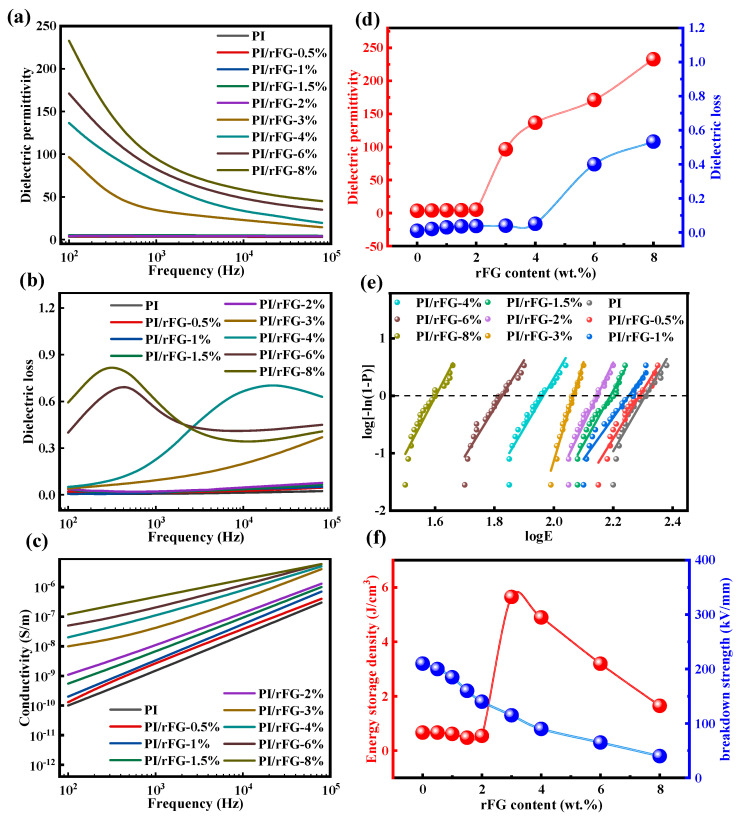
Dielectric property of PI/rFG composites films. (**a**) Curves of dielectric permittivity of the PI/rFG composite films with frequency; (**b**) curves of dielectric loss of the PI/rFG composite films with frequency; (**c**) curves of conductivity of the PI/rFG composite films with frequency; (**d**) dielectric permittivity and dielectric loss of the various PI/rFG composite films at 100Hz; (**e**) the Weibull distribution of dielectric breakdown strength of the PI/rFG composite films; (**f**) energy storage density and breakdown strength of the PI/rFG composite films.

The thermal performance of the PI/rFG composite films with high dielectric properties is an important requirement in practical applications. In this work, the thermal properties of the PI/rFG composite films were analyzed by TGA and DMA. As shown in Figure 3a,b and Table 1, the pure PI started to decompose at 450 °C, decomposing by 5% at 562.3 °C and remaining at 61.27% at 800 °C. Compared to the pure PI, the thermal performance of the PI/rFG composite films is slightly reduced. During the imidization process, the PEG inside the PI did not completely escape. Along with the increasing temperature, the PEG will continue to decompose and escape from the PI matrix, resulting in a slight decrease in the heat resistance of the PI. Figure 4b and Table 1 show the DMA curves and the glass transition temperature of the PI/rFG composite films with different proportions of FG–PEG added in the PI matrix. The glass transition temperature of the pure PI was measured as 284.1 °C. With 8 wt. % proportion of FG–PEG added in the PI matrix, the glass transition temperature increased to 294.5 °C. Graphene in the PI matrix can effectively block the movement of PI molecular chains, thus increasing the glass transition temperature of the PI.

In addition to excellent thermal properties, PI composites also need to have superior mechanical properties. As can be seen from Figure 3c,d and Table 1, the mechanical properties of PI are promoted. With the increasing proportion of rFG in the PI, the tensile strength of the PI/rFG composite films increases at first and then decreases. The tensile strength of pure PI was measured to be 159.7 MPa, and when the proportion of FG–PEG was added in the amount of 1 wt. %, the tensile strength of the PI composite films reached a maximum of 183.5 MPa, which was 15% higher than the pure PI, and the tensile strength of the PI/rFG composite films would gradually decrease if the proportion of FG–PEG added continued to increase. Moreover, the modulus of the PI/rFG films also improved in correlation with the proportion of FG–PEG added. PI/rFG composite films maintain a good mechanical strength and flexibility. By in-situ reduction of FG in the PAA composites, rFG is uniformly distributed in the PI matrix. When the PI/rFG composite films are stressed by external forces, the interaction between the rFG and PI chain segments will lead to an increase in tensile strength. When the proportion of rFG is too high, the rFG will agglomerate in the PI matrix, resulting in uneven stress loading in the films, so the tensile strength and elongation at the break of the PI composite films are reduced.

### 2.3. Dielectric Properties

The dielectric permittivity, dielectric loss and conductivity of the PI/rFG composite films are shown in Figure 4a–c and Table 2. As for PI, the dielectric permittivity at 100 Hz is 3.47 and the dielectric loss is 0.009. Furthermore, Figure 4d demonstrates the positive correlation between the dielectric permittivity of the PI/rFG composite films and the addition of FG–PEG. With 8 wt. % of FG–PEG added, the dielectric permittivity of the PI/rFG composite films reaches 235.74, which is 68 times higher than the pure PI, while the dielectric loss remains at 0.534. This result could be attributed to the fact that the dielectric permittivity of the dielectric material is closely related to the polarization. Graphene with good electrical conductivity is embedded in the PI matrix, forming numerous micro-capacitor structures. A strong polarization effect is formed between the numerous micro-capacitor structures under the action of the electric field. The increase in interfacial polarization due to the higher proportion of FG–PEG added leads to a corresponding rise in the dielectric permittivity of the PI/rFG composite films. Because of polarization, charged particles can overcome the energy loss caused by thermal motion under the influence of an electric field force, so there is a corresponding increase in dielectric loss. In different proportions of rFG, because the interface polarization occupies the main position in the low frequency band and the directional polarization occupies the main position in the high frequency band, the change trend for dielectric permittivity and dielectric loss of the PI/rFG composite films with frequency are different [58].

The breakdown strength of the PI/rFG composite films was analyzed through the Weibull distribution as shown in Figure 4e. A total of 20 points per film were tested separately for electrical breakdown and then calculated using Equations (1)–(3)

(1) $$P=1-\exp\left[-{\left(\frac{E}{E_{0}}\right)}^{\beta }\right]$$

(2) $$P_{i}=\frac{i-0.50}{n+0.25}$$

(3) $$\log\left[-\mathrm{In}\left(1\;-\;P\right)\right]=\beta \mathrm{logE}-{\beta \mathrm{logE}}_{0}$$

where *E* is the measured breakdown strength; *P* is the cumulative probability of the electrical failure; β is the shape parameter describing the scatter of the data; *i* is the number of ordering *E* from the smallest to the largest; and *n* is the number of all samples. The breakdown strength of the PI/rFG composite films reduces along with the proportion of rFG increasing. In the effect of a strong electric field, rFG and residual PEG in the PI composite films leads to a decrease in the breakdown strength.

The energy storage density of the PI composite film is calculated by the dielectric permittivity and dielectric loss (W = 0.5ℇ_0_ℇE^2^, ℇ_0_ = 8.854 × 10 ^−12^ F/m). As Figure 4f and Table 2 show, the energy storage density of the PI/rFG-8% composite films reached a maximum value of 5.651, which is 9 times higher than the pure PI films (0.664). With different particles as fillers, the dielectric properties of PI could be adjusted to various values in a wide range. In contrast, with FG–PEG as fillers, the dielectric properties of PI are significantly improved, as shown in Table 2.

## 3. Experimental

### 3.1. Materials

Biphenyltetracarboxylic dianhydride (BPDA, 99.5%, Chinatech Chemical Co., Ltd., Tianjin, China), oxydianiline (ODA, 99.0%, Chinatech Chemical Co., Ltd., Tianjin, China), dimethylacetamide (DMAc, AR, Sinopharm Chemical Reagent Co., Ltd., Shanghai, China), FG (F/C = 1.05, Hubei ZHUOXI Fluorochemical Co., Ltd., Hubei, China), and polyethylene glycol (PEG, MW: 400, AR, Wuxi Yatai United Chemical Co., Ltd., Shanghai, China).

### 3.2. Preparation of FG/PI Composite Films

**Scheme 1:**
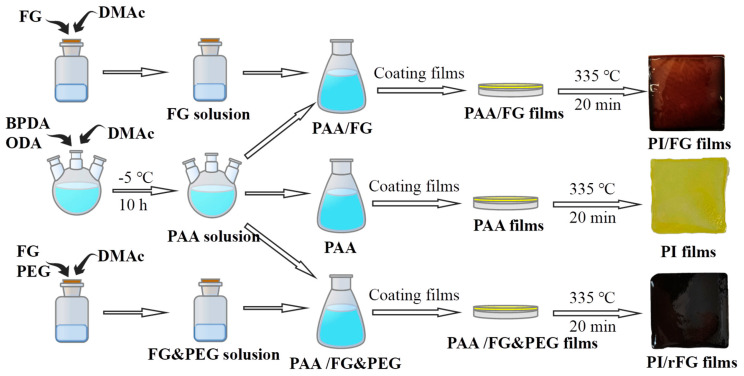
Preparation processes of various PI composite films.

A total of 2 g of FG and 8 g of PEG were added into 90 g of DMAc, and then the mixture was sonicated to achieve the uniform dispersion of FG (300 W, 30 min). PEG was added to promote the dispersion of FG in DMAc and reduce the FG to graphene (rFG). The PAA was obtained by reacting equimolar amounts of BPDA and ODA at −5 °C for 10 h. The FG–PEG solution and PAA were mixed together with different contents by magnetic stirring (1 h). After coating, all the casting films were thermally treated at 335 °C for 20 min to obtain PI/rFG films. As a comparison, pure PI films and PI/FG films without PEG were prepared simultaneously. According to the weight contents of the FG in PAA, the resultant composite film samples were named as PI, PI/rFG-0.5%, PI/rFG-1%, PI/rFG-1.5%, PI/rFG-2%, PI/rFG-3%, PI/rFG-4%, PI/rFG-6% and PI/rFG-8%, respectively. All the preparation processes are shown in Scheme 1.

### 3.3. Characterizations

The morphology was characterized by scanning electron microscopy (SEM, TESCAN CLARA, Liestal, Switzerland). An energy dispersive spectrometer (EDS, TESCAN CLARA, Liestal, Switzerland) was used to analyze the elements. The molecular structure was characterized by Fourier transform infrared spectra (FT-IR, TENSOR-27, China) at the wave number ranges from 400 cm^−1^ to 2800 cm^−1^. An LCR digital bridge (TH2819A, China) was applied to test the dielectric properties. In order to avoid undesired resistance and capacitance, all of the samples were adhered with silver conductive (7.0 cm × 7.8 cm) on two sides. The thermal properties were characterized by thermogravimetric analysis (TGA, TGA-55, Perkin-Elmer, Waltham, MA, USA) under a N2 atmosphere at a heating rate of 10 °C/min. Dynamic mechanical analysis (DMA, PerkinElmer Diamond, Perkin-Elmer, USA) was used to characterize the glass transition temperature of the PI composite films under a normal air atmosphere at a heating rate of 10 °C/min. The mechanical properties were analyzed by an electronic testing machine (CMT8102, Shenzhen, China) with a stretching rate of 10 mm/min.

## 4. Conclusions

PI with excellent mechanical properties and heat resistance has been widely applied in the field of microelectronics, and in order to correspond to the rapid development of the industry, PI/rFG composite films with ultra-high dielectric properties were fabricated by introducing graphene into the PI matrix through the method of in-situ reduction of FG. PI/rFG composite films significantly improved the dielectric properties of PI without losing mechanical properties and thermal properties. The dielectric permittivity of pure PI is 3.47 and the maximum energy storage density is 0.664 J/cm3. The maximum dielectric permittivity of the PI composite films in the same condition is 235.74, which is 68 times higher than pure PI, and the energy storage density is 5.651, which is 9 times higher than that of pure PI films.

## Figures and Tables

**Table 1:** The thermal and mechanical properties of the PI/rFG composite films.

Sample	T-5% (°C)	Residue-800 °C	T_g_ (°C)	Stress (MPa)	Strain	Modulus (GPa)
PI	562.3	61.27%	284.1	159.7	8.2%	3.6
PI/rFG-0.5%	560.3	60.54%	284.5	168.4	8.1%	3.8
PI/rFG-1%	557.1	59.85%	285.3	183.5	7.4%	4.4
PI/rFG-1.5%	555.5	59.06%	287.4	178.5	6.9%	4.8
PI/rFG-2%	550.7	59.36%	288.7	175.9	5.4%	5.5
PI/rFG-3%	545.5	59.13%	289.3	170.2	4.6%	5.8
PI/rFG-4%	541.3	58.75%	291.1	155.3	3.6%	6.1
PI/rFG-6%	534.5	58.27%	293.2	145.8	3.1%	6.5
PI/rFG-8%	530.1	57.88%	294.5	130.1	2.3%	7.3

**Table 2:** The dielectric properties of various PI composite films.

Filler	Filler Content	Dielectric Permittivity	Dielectric Loss	Breakdown Strength (kV/mm)	Energy Storage Density (J/cm^3^)	Ref.
PSF	40 wt. %	6.40	0.015	152	0.64	[33]
PVDF	50 wt. %	8.85	0.018	-	-	[34]
MOF	20% wt. %	8.80	0.034	208	0.39	[31]
Ag@SiO_2_	50 vol. %	11.70	0.015	-	-	[39]
BaTiO_3_	50 vol. %	29.66	0.009	59.5	0.465	[28]
LiTFSI	30 vol. %	38.18	1.600	42	0.30	[59]
GO	1 wt. %	68.00	0.600	-	-	[60]
MWCNTs	20 vol. %	217	1.580	45	1.957	[38]
CCTO	16.4 vol. %	171.00	0.450	-	-	[27]
rFG	0 vol. %	3.47	0.009	210	0.664	This work
	0.5%	3.77	0.019	200	0.663	
	1%	4.06	0.028	185	0.616	
	1.5%	4.19	0.035	160	0.476	
	2%	5.11	0.037	140	0.543	
	3%	96.50	0.040	115	5.651	
	4%	136.54	0.052	90	4.897	
	6%	171.02	0.410	65	3.198	
	8%	235.74	0.534	40	1.648	
